# Artificial Intelligence for Patient-Reported Outcomes in Oncology: Current Applications and Future Directions Toward Multimodal Monitoring

**DOI:** 10.3390/cancers18121905

**Published:** 2026-06-11

**Authors:** Sebastian Gorecki, Aleksandra Tatka, Malgorzata Osmola

**Affiliations:** Maria Sklodowska-Curie Medical Academy, Al. Solidarnosci 12, 03-411 Warszawa, Polandmalgorzata.osmola@uczelniamedyczna.com.pl (M.O.)

**Keywords:** artificial intelligence, patient-reported outcomes, oncology, machine learning, natural language processing, digital biomarkers, multimodal monitoring, EORTC QLQ-C30, PROMIS, PRO-CTCAE

## Abstract

Patient-reported outcomes (PROs) are an integral component of contemporary oncology. They provide direct insight into symptom severity, treatment tolerability, and health-related quality of life. Despite their clinical relevance, routine implementation faces several hurdles. Key limitations include patient survey fatigue, challenges in real-time interpretation of complex symptom trajectories, and incomplete longitudinal data that limit reliable analysis. This narrative review summarizes recent advances (2020–2026) in applying artificial intelligence (AI) to structured questionnaires, including EORTC QLQ-C30, PROMIS, and PRO-CTCAE, as well as to unstructured clinical text. Machine learning and natural language processing may enhance the clinical utility of PROs through automated analysis, symptom extraction, and predictive modeling. Current studies suggest that AI-based approaches can support the prediction of symptom deterioration, treatment-related toxicity, and healthcare utilization, including unplanned hospitalizations and emergency department visits. Furthermore, NLP models can extract clinically meaningful information from free-text narratives. We also discuss emerging non-invasive digital biomarkers derived from speech and facial expressions. Multimodal approaches suggest that these features may provide complementary indicators of pain, fatigue, and affective state. Overall, AI has the potential to transform PROs from static assessment tools into dynamic clinical instruments. This shift may enable more continuous and proactive symptom monitoring and support the integration of multimodal patient data into oncology decision-making workflows.

## Simple Summary

Cancer treatment often causes symptoms that affect quality of life and may change between clinical visits. Patient-reported outcomes allow patients to report symptoms directly, but large amounts of data can be difficult to interpret in real time. Artificial intelligence offers new opportunities to analyze patient-reported outcomes, identify symptom patterns, and predict clinical deterioration. This review summarizes recent developments in artificial intelligence methods applied to patient-reported outcomes in oncology, including machine learning, natural language processing, and emerging digital biomarkers derived from speech and facial expressions. We also discuss future multimodal monitoring approaches that combine questionnaire data with additional patient-generated information. These technologies may support earlier detection of complications, improve symptom management, and help healthcare professionals deliver more personalized cancer care.

## 1. Introduction

Oncological treatment places patients under a dual burden: the toxicities of therapy and the symptoms of the disease itself. The current standard of care in oncology largely relies on history-taking, which seems insufficient. Visits are often too infrequent, patients do not always report symptoms, and clinicians may overlook. This leads to a decreased quality of life for patients and can affect treatment adherence, resulting in worse outcomes. Patient-reported outcomes (PROs), defined as any report of a patient’s health status that comes directly from the patient without interpretation by a physician, can help address this gap by allowing systematic, structured and more frequent monitoring of symptoms directly from the patient’s perspective, including between visits.

PROs are vital to modern cancer care, which is shifting toward patient-centered models that prioritize quality of life and long-term health outcomes. An additional important feature of PROs is the collection of real-world data from patients’ experiences. These outcomes serve as the gold standard for capturing the subjective burden of cancer and its treatments. Reporting treatment toxicities, as well as quality of life, nowadays usually with PROS, is an important endpoint of clinical trials [1]. Currently, PROs’ results are increasingly treated as operational clinical signals and critical prognostic indicators in routine practice [2,3,4].

The prognostic importance of systematic monitoring of these outcomes is increasingly supported by evidence. Systematic reviews and meta-analyses investigating routine monitoring in adult oncology patients demonstrate measurable clinical benefits. Data show that the usage of PROs is an independent prognostic factor for overall survival across cancer populations [5]. These include a pooled 1-year mortality risk of 0.77 and a significant reduction in emergency room visits [6]. Survival advantage is largely attributed to the early detection of symptomatic adverse events, which captures domains that are difficult to assess in routine care [4]. These include pain interference, fatigue, and affective distress.

Despite its clinical utility, routine implementation still faces substantial logistical, cognitive, and organizational barriers that affect patients, caregivers, and clinicians. Traditional paper surveys often fail to capture daily fluctuations in symptoms. On the other hand, high-density surveys often induce survey fatigue, leading to diminished patient compliance, missing data, and sparse reporting trajectories [7,8].

Additionally, real-time interpretation of multidimensional PRO trajectories may increase clinician workload. Consequently, symptom management often remains reactive, initiated only when distress reaches a critical threshold [9].

Artificial intelligence, encompassing machine learning, deep learning, and natural language processing, provides a computational framework to bridge the gap between data collection and clinical action. The integration of artificial intelligence into oncology is advancing beyond diagnostic imaging and genomics to encompass the digital phenotyping of the patient experience [10]. Computational models may convert static questionnaires into predictive digital tools by summarizing complex multidimensional data into clinically interpretable risk estimates. This shift toward predictive oncology enables algorithms to forecast potential complications such as immunotherapy-related adverse events or unplanned hospitalizations [11]. At the same time, natural language processing and large language models can be used to analyze unstructured clinical text, including electronic health records, clinician notes, and patient-generated free text. This may improve the identification of symptom burden, detect clinically relevant information not captured by structured instruments, and facilitate more timely clinical decision-making.

Despite these advances, a significant gap remains in integrating multimodal data streams into clinically interpretable and deployable systems. Existing approaches are typically limited to single-modality analyses, with insufficient evidence on how to effectively fuse and validate combined structured, textual, and biometric data in real-world oncology settings. Unlike prior studies that focus on single-modality analyses, this review introduces a unified conceptual framework for the multimodal integration of structured PROs, unstructured clinical text, and digital biomarkers, highlighting their combined potential for early detection of masked distress and proactive clinical intervention.

Importantly, in this review, structured patient-reported outcomes are considered the primary clinical signal, while complementary modalities such as speech analysis, facial micro-expressions, passive sensing, and unstructured clinical narratives are discussed as auxiliary digital biomarkers that may enhance contextual interpretation of symptom burden rather than replace PROs themselves.

This narrative review synthesizes recent literature published between 2020 and 2026 and explores the evolving landscape of artificial intelligence applications for patient-reported outcome analysis in oncology. We focus on three primary domains. First, we examine the computational enhancement of structured instruments, including the EORTC QLQ-C30, PROMIS, and PRO-CTCAE. Second, we review the extraction of symptom data from unstructured clinical notes via natural language processing. Third, we highlight the emergence of non-invasive digital biomarkers derived from vocal acoustic features and facial expression patterns [12,13]. To address the limitations of isolated data streams, we propose the Multimodal PRO Analyzer, which integrates structured questionnaires with audiovisual biomarkers to detect masked distress, generate risk scores, and support clinical alerts. We also discuss key challenges, including interpretability [14,15], algorithmic bias, and regulatory considerations [16,17].

## 2. Materials and Methods

This manuscript represents a structured narrative review of the scientific, technical, and clinical literature concerning artificial intelligence and patient-reported outcomes in oncology, with a primary focus on studies published between 2020 and 2026. Although this work does not constitute a formal systematic review or meta-analysis, selected PRISMA-inspired elements were incorporated to improve methodological transparency, including explicit reporting of the search strategy, study screening process, and inclusion criteria. Due to the heterogeneity of included studies, datasets, clinical settings, and AI methodologies, quantitative synthesis was not considered appropriate.

The year 2020 was selected as the primary starting point because it marked a major acceleration in digital oncology associated with the COVID-19 pandemic, broader implementation of remote monitoring strategies, and the rapid emergence of large language models and digital biomarkers in clinical research. Nevertheless, selected landmark studies published before 2020 were additionally included to provide historical and methodological context for the development of AI-assisted PRO monitoring frameworks.

### 2.1. Search Strategy and Data Sources

The final literature search across all databases was conducted on 28 March 2026.

The databases included PubMed/MEDLINE, Web of Science, Scopus, and IEEE Xplore. Preprints from arXiv were consulted only for contextual and technical background and were not included as core clinical evidence. To reduce the risk of missing relevant publications, particularly studies published during the COVID-19 period or incompletely indexed articles, supplementary manual citation tracking and targeted Google Scholar searches were additionally performed.

### 2.2. Search Terms and Inclusion Criteria

Publications were included if they: (i) focused on oncology populations; (ii) investigated artificial intelligence, machine learning, deep learning, or natural language processing approaches; (iii) analyzed patient-reported outcomes, symptom monitoring, digital biomarkers, or multimodal monitoring strategies; (iv) were published in English between January 2020 and February 2026.

Studies unrelated to oncology, duplicate records, conference abstracts lacking methodological detail, purely technical engineering papers without clinical relevance, and articles focused exclusively on imaging or genomics without patient-centered outcomes were excluded.

Search queries combined medical and computational terminology using Boolean operators. The main keywords included: *“artificial intelligence”*, *“machine learning”*, *“deep learning”*, *“natural language processing”*, *“patient-reported outcomes”*, *“PROMs”*, *“EORTC QLQ-C30”*, *“PRO-CTCAE”*, *“oncology”*, *“cancer”*, *“digital biomarkers”*, *“facial expression analysis”*, *“voice analysis”*, and *“multimodal monitoring”*.

Reference lists of key studies and reviews were manually screened to identify additional relevant publications. A total of 842 records were screened during the initial search. After removing duplicates, 615 titles and abstracts were assessed for relevance, and 124 full-text publications were reviewed.

Studies included in the final thematic synthesis were selected based on methodological relevance, clinical applicability, novelty of AI methodology, and relevance to multimodal oncology monitoring. The thematic distribution of included studies is summarized in Table 1.

**Figure 1:**
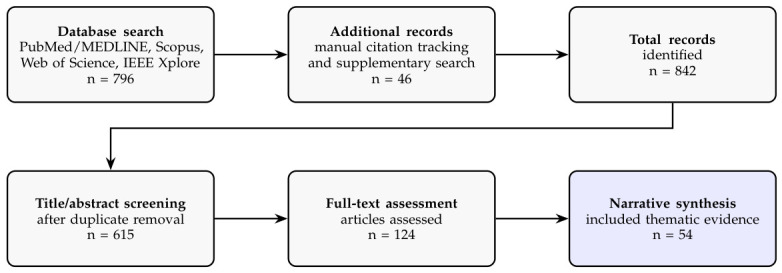
PRISMA-inspired workflow illustrating literature identification, screening, eligibility assessment, and thematic inclusion in this structured narrative review.

The reduction from 842 initially identified records to 124 full-text reviewed publications resulted primarily from duplicate removal, exclusion of non-oncology studies, exclusion of papers without direct relevance to PRO-related monitoring, removal of purely technical studies lacking clinical applicability, and exclusion of conference abstracts or reports without sufficient methodological detail. The distribution of records across individual literature sources is presented in Table 2. The overall study identification, screening, and eligibility assessment process is summarized in Figure 1.

## 3. Artificial Intelligence in the Analysis of Structured Patient-Reported Outcomes

Standardized patient-reported outcome instruments form the empirical foundation of subjective health assessment in oncology. These structured questionnaires are meticulously validated to capture the multifaceted impact of malignant disease and systemic therapies [18]. However, the complexity of questionnaires and the nonlinear relationships between data and symptoms require sophisticated analytical approaches to yield reliable predictive clinical insights. Machine learning algorithms are increasingly deployed to process these structured datasets, enabling the accurate forecasting of clinical trajectories [19].

### 3.1. Enhancing Predictive Modeling with the EORTC QLQ-C30

The EORTC QLQ-C30 is one of the most rigorously validated and widely utilized patient-reported outcome measures in global cancer research [20,21], translated to more than 60 languages, and validated for different diseases, well grounded for cross-cultural use [22,23]. While it provides high disease-specific granularity regarding a patient’s physical and psychosocial status, interpreting its multidimensional outputs to forecast long-term outcomes is mathematically complex.

Machine learning has transformed these tools from descriptive metrics into clinically useful prognostic tools. For instance, in gastrointestinal cancer populations, an algorithmically detected 10-point increase in physical functioning or global quality-of-life scales has been associated with an adjusted hazard ratio of 0.87, representing a 13% reduction mortality risk [24]. Machine learning models, such as random forests and extreme gradient boosting, enhance these predictions by capturing nonlinear interactions between quality of life domains and sociodemographic variables, providing a more nuanced risk stratification than traditional proportional hazard models [19,25].

Artificial intelligence is also widely used to map EORTC QLQ-C30 scores to preference-based health utility indices, such as the EQ-5D. In robust five-fold cross-validation studies, ridge regression demonstrated superior mapping performance, achieving an R-squared of 0.753, a root mean square error of 0.074, and a mean absolute percentage error of 8.2% [26]. Such algorithmic transformations provide a highly accurate alternative for estimating health utility values when preference-based measures are unavailable.

### 3.2. Toxicity Prediction and Acute Care Forecasting via PRO-CTCAE

The Common Terminology Criteria for Adverse Events (CTCAE) are a standardized system for clinician-reported classification of treatment-related adverse events. A complementary framework, the PRO-CTCAE, captures patient-reported symptomatic toxicities using structured questionnaires. Each symptom is graded according to standardized severity scales reflecting frequency, intensity, and functional interference. The system is widely used in oncological clinical trials and in everyday practice to assess patients’ symptoms and lab results to guide decisions.

While the EORTC QLQ-C30 provides a broad overview of general quality of life, the PRO-CTCAE was specifically engineered to capture granular symptomatic toxicities [27]. Predictive machine learning models have been trained on baseline health states, demographic data, and toxicity profiles to accurately predict selected toxicities associated with cancer treatments [25].

One of the most clinically impactful applications is the prediction of acute toxicities and unplanned hospitalizations. In patients undergoing neoadjuvant chemoradiotherapy for rectal cancer, predictive models incorporating gender, body mass index, and baseline emotional functioning achieved discrimination ability with an area under the curve of 0.687, identifying high-risk subgroups with a 6.4-fold increased likelihood of experiencing grade 3–4 toxicities [28].

In addition to standalone PRO-based models, recent approaches increasingly integrate patient-reported data with complementary sources of passive monitoring data. Furthermore, models that combine subjective data with passive monitoring, such as smartphone-derived daily step counts, have demonstrated promising predictive performance in forecasting acute care utilization [29,30,31]. In patients receiving systemic anticancer treatment, a decline in daily step counts, when processed through neural network models alongside survey data, can predict unplanned hospitalizations within the next 7 days, with an area under the curve of up to 0.88 [32]. These findings suggest that AI-assisted integration of passive monitoring and PRO data may support earlier intervention and reduce acute care utilization. This multimodal paradigm is consistent with broader approaches that combine PROs, electronic health records, and digital phenotyping to improve prediction of clinical outcomes.

### 3.3. Standardization, Adaptive Testing and Trajectory Mapping with PROMIS

The Patient-Reported Outcomes Measurement Information System (PROMIS) represents a major advancement in the psychometric standardization of subjective data. Designed to provide a common metric across disparate chronic diseases, the system utilizes item response theory and computerized adaptive testing. This dynamic testing serves as an advanced computational methodology that adjusts the sequence and difficulty of the questions presented based on the patient’s immediately preceding answers. This algorithmic approach drastically reduces the overall number of questions a patient must answer, actively mitigating survey fatigue.

In the rapidly evolving landscape of precision oncology, machine learning models frequently integrate these standardized domains, such as physical function, pain interference, and global mental health, as critical input features. For example, in evaluating patients undergoing highly complex cellular therapies for hematological malignancies, scores have been used alongside other data to accurately map the trajectory of physical and cognitive recovery post-infusion [33]. Algorithms processing these continuously collected metrics demonstrate the ability to track subtle longitudinal deviations from a baseline. This continuous surveillance allows for the early identification of signals of systemic decline that may precede overt clinical symptoms, thereby shortening the time to intervention in highly morbid treatment scenarios. A summary of major structured PRO instruments and their AI applications is presented in Table 3.

## 4. Natural Language Processing and Large Language Models in Unstructured Clinical Data

While structured questionnaires provide vital, quantifiable metrics, an expansive repository of nuanced, contextual patient experiences remains trapped within unstructured clinical text. Electronic health records, clinician progress notes, multidisciplinary tumor board summaries, and open-ended patient feedback contain complex narratives detailing symptom severity, psychosocial distress, and treatment response. Natural language processing (NLP) enables automated analysis of unstructured clinical text. It has emerged as a foundational tool to analyze unstructured clinical data, seamlessly transforming free text into structured, analyzable digital biomarkers [10,34].

### 4.1. Extracting Symptom Burden and Disease Response from Clinical Narratives

Clinical documentation in oncology is notoriously dense, frequently containing non-standardized abbreviations, implicit symptom relationships, typographical errors, and complex temporal timelines. Early rule-based natural language processing systems struggled immensely with the lexical complexity and semantic ambiguities of oncology narratives [34]. However, the advent of transformer architectures and large language models, such as Bidirectional Encoder Representations from Transformers, has substantially improved automated medical text extraction [35].

Unlike TF-IDF approaches, transformer models process the full contextual relationship between words within a sequence. This is achieved through the self-attention mechanism, mathematically defined in Equation (1), where *Q*, *K*, and *V* represent the query, key, and value matrices respectively, and $d_{k}$ denotes the scaling dimension factor:

(1) $$\mathrm{Attention}\left(Q,K,V\right)=\mathrm{softmax}\left(\frac{QK^{T}}{\sqrt{d_{k}}}\right)V$$

Although simplified, this mechanism illustrates how transformer-based models prioritize clinically relevant symptom expressions within long and complex oncology narratives, thereby improving automated extraction of symptom burden and treatment-related toxicity signals.

In the context of oncology, this mechanism enables models to selectively prioritize clinically relevant symptom expressions within complex patient narratives, improving the detection of subtle indicators of deterioration.

Beyond information extraction, natural language processing is increasingly used for direct outcome prediction. By analyzing free-text narratives at the start of chemotherapy, transformer-based models can predict the risk of acute care utilization. While models using structured health data typically achieve slightly higher performance (C-statistic 0.748, where values closer to 1 indicate better discrimination between patients with and without the outcome), NLP models using only language features from clinical notes have achieved highly comparable results (C-statistic 0.730). This demonstrates that linguistic signals in patient-reported narratives may serve as strong predictors of future health deterioration [36].

Furthermore, natural language processing combined with topic modeling, such as latent Dirichlet allocation, enables automated analysis of patient-reported experience measures and free-text survey comments. This provides healthcare systems with insights into patient concerns that are often missed by standardized numerical scales, such as subtle changes in psychological distress or specific barriers to medication adherence that may precede clinical relapse [37].

In oncological practice, self-attention-based natural language processing models are increasingly used to extract clinically relevant information from unstructured data, such as radiology reports and clinician notes. These models enable automated mapping of narrative descriptions to standardized response frameworks, including the Response Evaluation Criteria in Solid Tumors (RECIST). Importantly, this approach operates at the level of clinical text analysis and is distinct from digital biomarker extraction based on patient-derived physiological signals, such as voice or facial expressions, which are discussed in the following section. Deep learning–based NLP models have demonstrated high performance, with reported high performance under controlled validation settings, with accuracy values reaching 95–99% in selected structured extraction tasks; however, reported performance metrics varied substantially across datasets and evaluation protocols. Notably, these models analyze clinician-generated reports rather than raw imaging data and can outperform traditional rule-based methods while demonstrating performance comparable to expert-annotated reference standards in large-scale information extraction [38].

Beyond purely physiological outcomes, natural language processing is highly instrumental in evaluating psychosocial distress and body image perception, particularly in highly morbid oncology settings such as head and neck or upper gastrointestinal tract cancers [39]. Advanced algorithms parse transcribed patient narratives to conduct sophisticated sentiment analysis and continuously gauge the intensity of basic emotions. By isolating specific vocabulary patterns and syntactic structures, models can flag early indicators potentially associated with psychological distress or depressive symptomatology. However, it is important to note that the performance of NLP-based models is highly dependent on documentation quality, which varies significantly across institutions. Inconsistent terminology, missing context, and variability in clinical reporting may introduce noise and reduce model robustness in real-world settings [34,36,38].

### 4.2. Large Language Models and Retrieval Augmented Generation in Electronic Platforms

A critical systemic limitation of traditional electronic patient-reported outcome collection infrastructure is the absence of an immediate personalized feedback loop. Patients diligently submit their symptoms via a digital portal, but unless a predefined severity threshold is breached, triggering an alert, the data may remain unreviewed by a clinician until the next scheduled outpatient appointment (or even be completely omitted). This may affect patient engagement in PRO participation.

**Figure 2:**
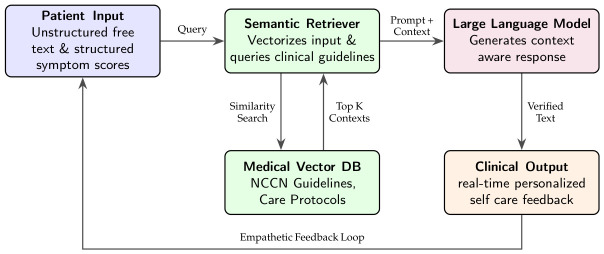
Workflow architecture of a Retrieval-Augmented Generation system in oncology. The system anchors patient-reported text to validated medical databases, reducing hallucination risk and supporting real-time, actionable self-care recommendations.

The integration of modern large language models with Retrieval-Augmented Generation architectures may support real-time, context-aware feedback generation for patients, as illustrated in Figure 2. In such systems, model outputs are grounded in verified medical knowledge bases, including institutional clinical protocols and oncology guidelines.

Despite their substantial potential, large language models remain vulnerable to hallucinations, clinically inaccurate outputs, hidden biases, and limited transparency of proprietary architectures. In oncology settings, such limitations may contribute to misinformation, inappropriate symptom interpretation, or overconfidence in automatically generated recommendations. Consequently, AI-generated outputs should not be interpreted as autonomous clinical decisions and must remain under clinician supervision. Additional concerns include reproducibility, explainability, and dependency on commercial closed-source systems. A prospective observational study recently examined the integration of such an architecture into a web-based platform for 42 patients undergoing highly toxic radiotherapy regimens for head and neck cancer [40]. Patients completed entries twice weekly, and the artificial intelligence generated immediate feedback based on the reported symptom profiles. Implementation of this feedback loop demonstrated a clinically meaningful effect: high engagement was directly correlated with significantly reduced body weight loss (4.45 percent versus 7.57 percent) and fewer treatment interruption days (0.67 versus 2.50 days).

Similarly, pilot applications using advanced language models to analyze longitudinal side-effect data from cancer patients over a four-week period effectively synthesized complex trajectories of quality of life, mood status, and pain management. These applications generated actionable clinical summaries, reducing the administrative burden on the nursing staff. By empowering patients with immediate, safe, and context-aware feedback, these integrated systems successfully transition from passive data-collection repositories into active AI-assisted supportive monitoring systems that improve patient resilience and adherence to oncological regimens [41].

The proposed architecture illustrates how AI-assisted systems may extend traditional symptom reporting with automated feedback and contextual risk stratification.

## 5. Digital Biomarkers and Multimodal Integration

Recent directions in precision oncology research increasingly involve the integration of non-invasive digital biomarkers. Recent research increasingly explores the integration of non-invasive digital biomarkers in precision oncology. An overview of major digital biomarker modalities and associated AI extraction methodologies is provided in Table 4. By capturing subconscious physiological signals, such as acoustic vocal variations and facial micro-expressions, artificial intelligence–assisted analysis may support the detection of masked distress and preemptively identify clinical deterioration. Despite their potential, digital biomarkers remain sensitive to external factors, including recording conditions, background noise, and inter-individual heterogeneity, which may limit reproducibility [10,42,43].

General-purpose frameworks such as OpenFace and OpenSMILE may demonstrate reduced robustness in uncontrolled clinical environments due to variability in illumination, camera positioning, microphone quality, background noise, demographic heterogeneity, accent variability, and partial facial occlusions. Consequently, domain-specific calibration and prospective clinical validation remain necessary before large-scale deployment in oncology settings.

### 5.1. Vocal and Acoustic Feature Extraction

Vocal features may reflect the physiological and psychological status of physical and psychological well-being. Variations in vocal cord tension, respiratory support, and articulatory precision are heavily influenced by systemic fatigue, pain, and neurological toxicity. The fundamental frequency, which reflects the rate of glottal opening and closing, is often altered in oncology patients [44]. More critically, the harmonic-to-noise ratio shows significant variability in malignant cases, indicating improper closure of the vocal cords. Studies using extensive datasets such as Bridge2AI Voice have confirmed that these acoustic markers can distinguish among healthy individuals, those with benign lesions, and those with laryngeal cancer with high statistical significance [45].

Vocal biomarkers also provide a clinically relevant window into the systemic and psychological impact of cancer treatment. Acoustic features have been associated with psychological distress and fatigue, reflecting phenomena such as flattening of prosody and increased vocal effort observed in patients with severe fatigue or psychomotor retardation.

Composite acoustic indices combining multiple vocal features have shown potential for detecting psychological distress, suggesting a non-invasive approach to monitoring symptom burden in oncology.

### 5.2. Facial Expression and Affect Analysis

In parallel with acoustic analysis, visual data streams derived from facial photographs or short video clips may provide clinically relevant insights into a patient’s affective state [43]. The automated recognition of pain and emotional distress is achieved by deploying deep convolutional neural networks [46]. These systems utilize the Facial Action Coding System, which deconstructs facial expressions into action units.

Decades of behavioral research have identified a core set of pain-related action units that have been consistently associated with pain-related facial responses. These include action unit 04 (brow lowerer), action units 06 and 07 (orbital tightening), action units 09 and 10 (mid-face levator contraction), and action unit 43 (eye closure). The dynamic intensity of these units is algorithmically combined to calculate the Prkachin Solomon Pain Index, a validated 16-point scale for pain intensity.

Traditional automated recognition systems often struggle in dynamic clinical environments like oncology wards [47]. However, recent advancements in deep learning, including Action Unit Region-based Convolutional Neural Networks, have substantially improved detection performance, achieving an F1 score of 0.77, a metric reflecting the balance between correctly identifying true signals and avoiding false detections, in critically ill populations. This significantly outperforms general-purpose tools such as OpenFace, which typically achieve an F1 score of approximately 0.42 under comparable conditions. Advanced weighted analytical frameworks that prioritize specific facial regions have demonstrated further performance gains, reaching up to 92.72% accuracy in estimating pain intensity, although different evaluation metrics were used across studies, thereby enabling more precise and timely analgesic interventions [48].

Crucially, systems like OpenFace leverage the Facial Action Coding System to quantitatively measure the intensity of specific facial muscle movements, known as Action Units. For instance, the activation of Action Unit 04 (brow lowerer), Action Unit 06 (cheek raiser), and Action Unit 12 (lip corner puller) is highly correlated with nociception and psychological distress. Algorithmic analysis of these micro-expressions enables the continuous estimation of facial stress, facial pain, and overall affect [13]. This objective visual assessment is particularly vital for patients who may underreport their symptoms due to cognitive impairment or poor performance status.

**Table 4:** Overview of non-invasive digital biomarkers and artificial intelligence extraction methodologies in oncology.

Biomarker Modality	Key Features Extracted	Computational Tools	Clinical Correlates
**Vocal and Acoustic**	Fundamental frequency, Mel frequency cepstral coefficients, jitter, shimmer, and harmonic-to-noise ratio [12,44,45].	Standardized acoustic feature extraction toolkits (e.g., OpenSMILE, librosa) and deep neural network–based models [12,45].	Systemic fatigue, vocal cord toxicity, respiratory distress, and mood alterations [12,44,45].
**Facial micro-expressions**	Facial Action Coding System units including Action Unit 04 (brow lowerer) and Action Unit 06 (cheek raiser) [43,46,48].	OpenFace, DeepFace, and specialized convolutional neural networks [46,47,48].	Nociception, acute pain exacerbation, affective state, and cognitive load [46,47,48].
**Multimodal Fusion**	Concatenated audiovisual feature vectors integrated with structured patient-reported data [10,19,49].	Early and late fusion architectures, including gradient-boosting models [49].	Detection of masked distress, automated triage, and composite risk stratification [10,19,49].

### 5.3. Proposed Architecture: The Multimodal PRO Analyzer

**Figure 3:**
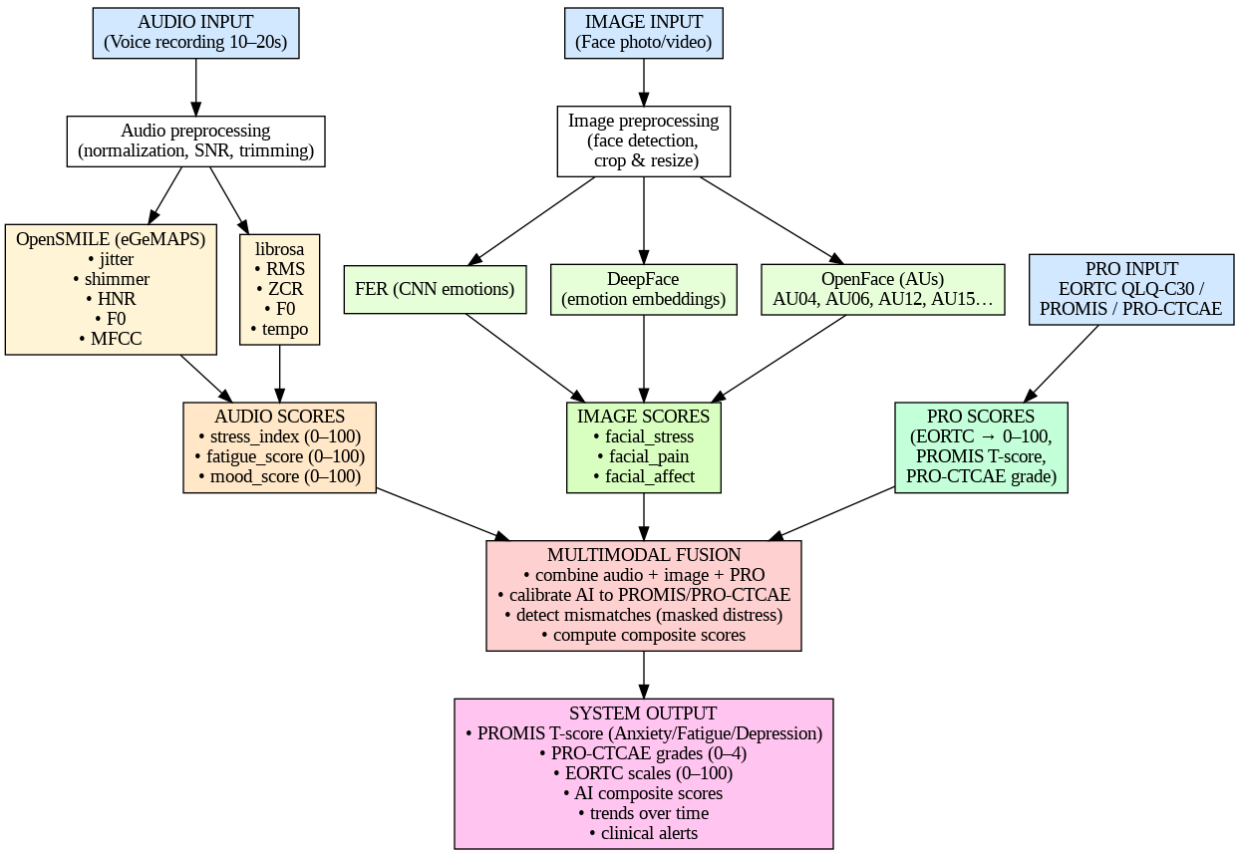
Block diagram of the proposed Multimodal PRO Analyzer architecture. The framework integrates acoustic feature extraction, facial micro-expression analysis, and structured clinical questionnaires within a multimodal fusion pipeline to generate calibrated composite risk scores, detect masked distress, and support real-time clinical alerts.

To bridge the gap between isolated digital biomarkers and established clinical workflows, we propose a novel conceptual architecture: the Multimodal PRO Analyzer (Figure 3). This integrated framework synthesizes three parallel data streams to provide an integrated real-time assessment of patient status.

Importantly, the proposed Multimodal PRO Analyzer should be interpreted as a conceptual translational framework intended to illustrate future integration pathways rather than a clinically validated diagnostic or decision-support system. The proposed conceptual architecture illustrates a potential integration framework combining audio and visual inputs, as well as structured patient-reported outcome data, such as responses to the EORTC QLQ-C30. The core innovation of this system lies in its multimodal fusion module. This central component concatenates the derived audio scores, image scores, and structured survey results into a unified high-dimensional feature space. The artificial intelligence engine calibrates these combined signals against standardized metrics like PROMIS T scores and PRO-CTCAE grades.

A critical function of this fusion is the detection of masked distress, identifying instances where a patient reports low symptom burden on a questionnaire, but their acoustic and facial biomarkers indicate severe pain or emotional fatigue. The ultimate system output provides a comprehensive composite clinical profile. This includes standardized clinical grades, artificial intelligence-derived composite scores, longitudinal trend analysis, and automated clinical alerts, thereby supporting a transition from reactive symptom management toward more proactive monitoring strategies. This architecture illustrates how multimodal inputs may be transformed into clinically actionable outputs through sequential feature extraction and fusion.

From a computational perspective, the fusion module may operate under different strategies, including early fusion (feature-level concatenation), late fusion (decision-level aggregation), or hybrid approaches combining both paradigms. Early fusion enables the model to learn cross-modal interactions between acoustic, visual, and questionnaire-derived features, while late fusion allows independent modality-specific models to contribute weighted predictions to the final risk score. The selection of fusion strategy depends on data availability, modality reliability, and clinical context. An additional challenge involves inter-modality heterogeneity, where differences in temporal resolution, signal reliability, and missingness patterns may substantially affect fusion stability and downstream clinical prediction performance. In real-world clinical deployment, multimodal systems must address challenges related to missing modalities, asynchronous sampling frequencies, noisy recordings, and incomplete longitudinal trajectories. Potential mitigation strategies include modality-aware inference, confidence-weighted fusion, temporal alignment algorithms, missing-data imputation, and uncertainty estimation mechanisms. Furthermore, audio and visual biomarkers may be substantially influenced by environmental recording conditions, requiring robust preprocessing and quality-control pipelines.

In addition to cross-sectional analysis, the system is designed to operate on longitudinal patient data streams. Temporal modeling techniques, such as recurrent neural networks, temporal convolutional networks, or transformer-based sequence models, can capture trends, fluctuations, and deviations from individual patients’ baselines over time.

The composite risk score is derived through weighted integration of modality-specific outputs, where each signal is normalized and calibrated against clinically validated scales such as PROMIS T-scores and PRO-CTCAE grades. This calibration ensures that AI-derived outputs remain interpretable within established clinical frameworks.

To ensure robustness and clinical reliability, the system may incorporate uncertainty estimation mechanisms, such as confidence scoring or Bayesian inference, allowing clinicians to assess the reliability of predictions generated from heterogeneous and potentially noisy multimodal inputs.

Additionally, explainability modules may be integrated into the architecture to provide feature-level attribution, enabling clinicians to understand which modality or specific features contributed most significantly to the generated risk score.

In practical terms, this architecture is intended to support earlier recognition of clinically relevant deterioration, particularly in situations where subjective questionnaire responses may underestimate symptom burden.

#### Clinical Benefits and Risks of the Multimodal PRO Analyzer

The proposed Multimodal PRO Analyzer offers several clinically relevant advantages over traditional single-modality monitoring systems.

From a clinical perspective, the primary benefit lies in enhanced sensitivity to early symptom deterioration. By integrating structured questionnaires with objective digital biomarkers, the system enables the detection of masked distress, where patient-reported symptoms underestimate the true physiological or psychological burden. This may facilitate earlier intervention, reduce unplanned hospitalizations, and improve treatment adherence.

Furthermore, multimodal integration supports more robust risk stratification by combining complementary data sources, thereby reducing reliance on any single, potentially biased modality. The inclusion of longitudinal modeling enables continuous monitoring and dynamic adaptation to individual patient trajectories, which is critical in complex oncology pathways.

The implementation of such systems is associated with several challenges. First, multimodal models introduce increased computational and infrastructural complexity, which may limit scalability in resource-constrained clinical settings. Second, variability in data quality—particularly in audio and visual inputs—may affect model reliability and reproducibility. Third, the integration of heterogeneous data sources raises concerns regarding interpretability, thus requiring advanced explainability mechanisms to ensure clinical trust.

In addition, the use of biometric data, such as facial expressions and vocal signals, raises significant ethical and regulatory considerations, particularly regarding patient privacy, data protection, and informed consent. Finally, the risk of alert fatigue remains a critical issue, necessitating careful calibration of alert thresholds and integration into existing clinical workflows.

Overall, while the Multimodal PRO Analyzer offers a promising framework for next-generation patient monitoring, its clinical adoption will depend on rigorous validation, robust governance, and seamless integration into healthcare systems.

## 6. Evidence Synthesis of AI Applications in PRO Analysis

The included studies demonstrate a clear trend toward integrating structured patient-reported outcomes, unstructured clinical data, and emerging digital biomarkers, highlighting the transition to multimodal predictive oncology. Representative studies included in this review are summarized in Table 5.

Overall, the reviewed studies demonstrate consistent improvements in predictive performance when integrating multiple data modalities. However, prospective validation remains limited, and most evidence is derived from single-center or retrospective cohorts.

## 7. Clinical Integration Pathway

**Figure 4:**
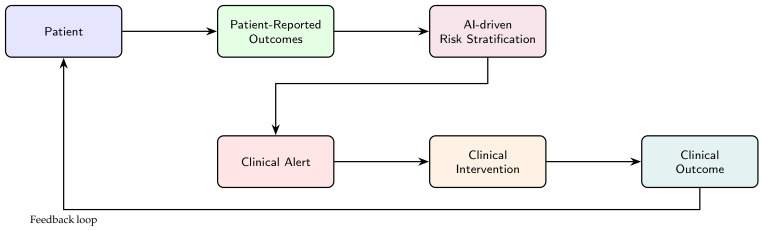
Clinical integration pathway of artificial intelligence-enhanced patient-reported outcomes. The framework illustrates the transformation of patient-reported data into AI-driven risk stratification, clinical alerts, and targeted interventions, which forms a continuous feedback loop that enables adaptive and personalized oncology care.

To translate patient-reported outcomes enhanced by artificial intelligence into clinical practice, a structured integration pathway is required. Figure 4 illustrates the conceptual workflow from patient input to clinical outcome.

The proposed clinical pathway illustrates how static symptom reporting may evolve into a more dynamic decision-support process. By linking patient-reported data with artificial intelligence–driven analysis and actionable clinical interventions, the pathway highlights the transition from reactive to proactive oncology care. From a clinical perspective, this pathway reflects a continuous monitoring loop rather than a one-time assessment. Patients provide symptom data through structured questionnaires or passive digital biomarkers, which are continuously analyzed by AI models. When clinically significant deviations from baseline are detected, automated alerts are generated and routed to healthcare providers. This enables early intervention before symptom escalation, shifting oncology care from reactive management to proactive, data-driven decision-making.

Importantly, this pathway operates longitudinally, allowing repeated cycles of assessment and intervention, which enables dynamic adaptation to patient condition over time.

This conceptual pathway highlights the critical role of artificial intelligence as an intermediary layer that translates subjective patient input into objective, actionable clinical signals.

## 8. Clinical and Regulatory Discussion

The clinical translation of artificial intelligence–enhanced patient-reported outcomes requires careful consideration of model performance, interpretability, workflow integration, and regulatory compliance. Importantly, improved predictive performance does not necessarily translate into improved clinical outcomes. Prospective interventional studies remain necessary to determine whether AI-assisted monitoring systems can meaningfully improve survival, quality of life, or healthcare utilization in routine oncology practice.

### 8.1. Multimodal Fusion Architectures and Performance Gains

Multimodal fusion approaches integrating structured PROs with digital biomarkers demonstrate improved predictive performance compared to unimodal models, supporting their relevance for clinical risk stratification [49].

### 8.2. Explainability and the Black Box Dilemma

Model interpretability remains a prerequisite for clinical adoption, necessitating the use of explainable artificial intelligence techniques to ensure transparent and clinically meaningful predictions [50]. Shapley Additive Explanations, grounded in game theory, enable feature-level attribution by quantifying the contribution of individual variables to model outputs; for example, such methods can demonstrate that changes in patient-reported emotional functioning may exert a stronger influence on survival predictions than selected imaging-derived features, thereby improving clinical interpretability and trust [51].

### 8.3. Algorithmic Bias and Regulatory Compliance

Algorithmic bias related to demographic and phenotypic variability must be actively mitigated through diverse training datasets and bias-aware modeling strategies to ensure equitable performance across patient populations [52,53].

### 8.4. Clinical Workflow Integration and Alert Fatigue

Effective integration into clinical workflows requires minimizing alert fatigue through adaptive thresholding and personalized baseline modeling [54].

### 8.5. Regulatory Classification as Software as a Medical Device

Artificial intelligence systems influencing clinical decision-making are increasingly regulated as Software as a Medical Device, requiring prospective validation and lifecycle management [55]. A critical challenge in this context is algorithmic drift, which refers to performance degradation over time due to changes in patient populations or clinical practices; regulatory frameworks such as Predetermined Change Control Plans aim to address this issue by enabling controlled model updates within predefined safety boundaries [56].

### 8.6. The European Union AI Act and Data Governance

Compliance with the European Union AI Act and data protection regulations necessitates robust governance frameworks, with federated learning representing a promising approach to privacy-preserving model development [16,17,57,58].

These challenges and corresponding technological and regulatory considerations are summarized in Table 6.

## 9. Limitations

This review has several important limitations. First, the manuscript represents a narrative rather than a systematic review, which may introduce selection bias despite the structured search strategy. Second, the included evidence remains highly heterogeneous with respect to patient populations, cancer types, modalities, datasets, and validation methodologies. Many artificial intelligence models discussed in this review were additionally trained on relatively small, institution-specific, or highly selected datasets, which may limit generalizability across broader and more diverse oncology populations.

Third, a substantial proportion of the reviewed studies were retrospective or exploratory, with relatively limited prospective multicenter validation. Some cited digital biomarker and multimodal studies were additionally derived from mixed or non-oncology populations, which may limit direct extrapolation to oncology-specific settings.

Additionally, external validation across geographically and demographically diverse oncology populations remains limited, and future studies should address potential dataset shift and institutional variability.

Furthermore, the proposed Multimodal PRO Analyzer represents a conceptual translational framework and has not undergone clinical validation, proof-of-concept implementation, or prospective deployment. Additional challenges include missing data, synchronization of heterogeneous modalities, variable recording quality, and regulatory barriers associated with biometric data processing.

Finally, the integration of large language models into oncology workflows remains associated with risks related to hallucinations, transparency limitations, model drift, and dependency on proprietary systems. Future research should prioritize prospective validation, explainability, robustness, and integration into real-world clinical workflows.

## 10. Conclusions

This review demonstrates that artificial intelligence may substantially expand the clinical utility of patient-reported outcomes in oncology by enabling more precise symptom monitoring, earlier detection of clinical deterioration, and more contextual interpretation of longitudinal patient data. The reviewed studies suggest that combining structured PROs with NLP and digital biomarkers may improve longitudinal symptom monitoring in oncology. Future research should prioritize prospective multicenter validation studies, standardized benchmarking datasets, and clinically interpretable multimodal architectures capable of integrating structured PROs, unstructured clinical narratives, and passive digital biomarkers. Additional priorities include explainability, uncertainty estimation, privacy-preserving learning strategies, and seamless integration into oncology workflows with minimal clinician burden. The development of robust translational pipelines and regulatory-ready validation frameworks will likely influence the future clinical adoption of AI-assisted PRO monitoring systems. However, most currently available evidence remains exploratory or retrospective, and prospective validation of multimodal systems in real-world oncology settings is still limited. Future progress in this field will require rigorous clinical validation, explainable model architectures, robust multimodal fusion strategies, and careful integration into existing healthcare workflows and regulatory frameworks.

## Figures and Tables

**Table 1:** Thematic distribution of studies included in the review.

Thematic Domain	Included Studies
Structured PRO analysis	39
Natural language processing and LLMs	31
Voice and acoustic biomarkers	18
Facial expression analysis	14
Multimodal fusion systems	22

Individual studies could contribute to multiple thematic domains; therefore, category totals are not mutually exclusive.

**Table 2:** Distribution of records identified during the literature search process across individual sources.

Database/Source	Records	Purpose and Scope
PubMed/MEDLINE	312	Primary biomedical and clinical oncology literature source focused on patient-reported outcomes, toxicity monitoring, and translational oncology studies.
Scopus	228	Broad interdisciplinary database covering medical artificial intelligence, digital health, and clinical informatics applications.
Web of Science	167	Supplementary citation-indexed literature source used to identify high-impact oncology and digital medicine publications.
IEEE Xplore	89	Technical and engineering-oriented publications related to machine learning, signal processing, multimodal fusion, and biomedical AI systems.
Manual citation tracking and supplementary search	46	Additional landmark studies identified through reference analysis, expert-guided screening, and supplementary Google Scholar searches.
Total	842	Records screened prior to eligibility assessment and duplicate removal.

The search strategy combined biomedical, interdisciplinary, and engineering-oriented databases with supplementary manual citation tracking to reduce the risk of omission of clinically relevant or emerging translational studies.

**Table 3:** Summary of structured patient-reported instruments and artificial intelligence integration in oncology.

PRO Instrument	Primary Domain Focus	Key AI/ML Applications	Predictive Outcomes and Clinical Utility
**EORTC QLQ-C30**	General quality of life, functional status, and core cancer symptoms.	Ridge regression mapping, principal component analysis, multiple linear regression, and k-means clustering.	Survival prediction, quality-of-life trajectories, and health utility estimation (e.g., EQ-5D mapping) [21,24,26].
**PRO-CTCAE**	Patient-reported symptomatic toxicities, including frequency, severity, and interference.	Random forest, latent profile analysis, and extreme gradient boosting classifiers.	Prediction of treatment-related toxicity, symptom burden modeling, and support for individualized treatment decisions [2,27].
**CTCAE**	Clinician-reported adverse events and toxicity grading based on standardized criteria.	Supervised learning models for toxicity prediction and clinical decision support systems.	Prediction of adverse events, treatment toxicity risk stratification, and validation of clinician-reported outcomes [25,28].
**PROMIS**	Standardized cross-disease health domains covering physical, mental, and social health.	Computerized adaptive testing and deep learning-based time-series modeling.	Longitudinal symptom monitoring, recovery trajectory modeling, and reduction of assessment burden [32,33].

Abbreviations: EORTC QLQ-C30—European Organization for Research and Treatment of Cancer Quality of Life Questionnaire Core 30; PRO-CTCAE—Patient-Reported Outcomes version of the Common Terminology Criteria for Adverse Events; CTCAE—Common Terminology Criteria for Adverse Events; PROMIS—Patient-Reported Outcomes Measurement Information System; EQ-5D—EuroQol 5-Dimension health status measure.

**Table 5:** Summary of key studies on artificial intelligence applications in patient-reported outcomes and multimodal monitoring in oncology.

Study	Modality	Cancer Type	Clinical Outcome	Validation
Diefenhardt et al. (2022) [28]	Structured PRO (EORTC QLQ-C30) + clinical variables	Rectal cancer	Prediction of acute toxicity (Grade 3 and 4)	Phase III trial cohort, internal validation
Brouwer et al. (2025) [32]	PRO + passive sensor data (step count)	Mixed solid tumors	Prediction of unplanned hospitalization (7-day horizon)	Prospective observational cohort
Fanconi et al. (2023) [36]	Unstructured clinical text (NLP)	Mixed oncology population	Acute care utilization risk prediction	Retrospective cohort, external comparison
Elbatarny et al. (2023) [38]	Radiology reports (NLP)	Metastatic cancer	Automated response classification (RECIST/OR-RADS)	Retrospective validation study; reported accuracy 95–99%
Liao et al. (2025) [40]	ePRO + LLM (RAG-based system)	Head and neck cancer	Weight loss reduction, treatment interruption days	Single-center prospective observational study (n = 42)
Liao et al. (2024) [41]	LLM-assisted symptom analysis	Mixed oncology population	Symptom trajectory summarization and decision support	Pilot clinical evaluation
Jenkins et al. (2025) [45]	Voice biomarkers (acoustic analysis)	Laryngeal cancer	Differentiation between benign and malignant lesions	Exploratory dataset study
Nerella et al. (2021) [48]	Facial action units (computer vision)	Critically ill patients	Pain detection (AU-based models)	Clinical dataset, F1-score evaluation
Flack et al. (2025) [49]	Multimodal fusion (clinical + biomarkers)	Pan-cancer	Survival prediction (C-index improvement)	Retrospective multi-cohort validation

**Table 6:** Translational challenges and proposed computational solutions for multimodal monitoring in clinical practice.

Domain	Clinical Challenge	Proposed Solution	Regulatory Considerations
**Interpretability**	In clinical practice, model outputs that lack clear explanation are difficult to trust and act upon. Clinicians need to understand which symptoms or signals contributed to a given prediction, especially when decisions affect treatment or hospitalization risk [50,51].	Use of explainability methods (e.g., SHAP, LIME) to highlight the contribution of individual features, such as specific PRO domains or biometric signals, to the final prediction.	The EU AI Act classifies such systems as high-risk and requires transparency, traceability, and the ability to explain model outputs to support clinical decision-making.
**Data Privacy**	Multimodal systems often rely on sensitive data, including voice recordings and facial images, which raise concerns about patient consent, data storage, and cross-border data transfer [16].	Adoption of federated learning or on-site data processing approaches, where raw patient data remain within the healthcare institution.	Strict requirements under the GDPR apply to biometric and health data, including explicit consent, data minimization, and secure processing. The EU AI Act further reinforces obligations for high-risk medical AI systems.
**Clinical Workflow**	Frequent alerts and false positives may overwhelm clinical teams, leading to alert fatigue and reduced responsiveness to truly critical events [54].	Personalized baseline modeling and adaptive thresholds that trigger alerts only when significant deviations from an individual patient’s trajectory are detected.	Regulatory frameworks emphasize risk management and human oversight, requiring systems to support—not replace—clinical decision-making and to minimize harm from over-alerting.

## Data Availability

The original contributions presented in this study are included in the article. Further inquiries can be directed to the corresponding author.

## Abbreviations

AI	Artificial Intelligence
AUC	Area Under the Curve
BERT	Bidirectional Encoder Representations from Transformers
EHR	Electronic Health Record
EORTC QLQ-C30	European Organization for Research and Treatment of Cancer
	Quality of Life Questionnaire Core 30
EU AI Act	European Union Artificial Intelligence Act
FDA	Food and Drug Administration
GDPR	General Data Protection Regulation
LLM	Large Language Model
ML	Machine Learning
NCCN	National Comprehensive Cancer Network
NLP	Natural Language Processing
PCCP	Predetermined Change Control Plan
PRO	Patient-Reported Outcome
PRO-CTCAE	Patient-Reported Outcomes version of the Common
	Terminology Criteria for Adverse Events
PROMIS	Patient-Reported Outcomes Measurement Information System
QALY	Quality Adjusted Life Year
RAG	Retrieval Augmented Generation
RECIST	Response Evaluation Criteria in Solid Tumors
SaMD	Software as a Medical Device
